# Supplementary material for: An Empirical Test of the Role of Value Certainty in Decision Making
Source: Front Psychol. 2020 Sep 18;11:574473. doi: 10.3389/fpsyg.2020.574473 (PMC7605174; doi:10.3389/fpsyg.2020.574473)
Supplement: Supplementary file 1 [file Data_Sheet_1.docx]

**Supplementary Material**

*Data Quality Check*

**Study 1**

Before testing our hypotheses, we performed a number of simple data quality checks. First, we assessed the test-retest reliability of value ratings. For each participant, we thus measured the correlation between first rating (Rating1) and second rating (Rating2), across items. We found that ratings were generally consistent (median Spearman’s rho = 0.817). Most participants showed a correlation of greater than 60%. We then measured, for each participant, the correlation between Rating1 and Rating3. We found that ratings were generally consistent (median Spearman’s rho 0.818).

Next, we performed a similar assessment of the test-retest reliability of certainty reports. Before examining the certainty data, we first converted the qualitative reports to numbers (“not at all” = 1, “slightly” = 2, “somewhat” = 3, “fairly” = 4, “very” = 5, “extremely” = 6). For each participant we then measured the correlation between certainty for Rating1 (Certainty1) and certainty for Rating2 (Certainty2), across items. We found that certainty reports were generally consistent (median Spearman’s rho = 0.352), although much less so than value ratings. We then measured, for each participant, the correlation between Certainty1 and Certainty3, across items. We found that certainty reports were generally consistent (median Spearman’s rho = 0.353), although much less so than value ratings.

Because rating certainty is a key variable for testing our hypotheses, we needed to be sure that participants responded meaningfully to the rating certainty question. An analysis of how certainty correlates with our other variables of interest would not be possible where there is insufficient variability in the certainty data. For this reason, we calculated a score for how much variance each participant had across certainty reports. The median certainty report variance was 0.935, 0.882, and 0.599 for Rating1, Rating2, and Rating3, respectively. We deemed that there were no participants who were obvious outliers based on this score.

For each of the test-retest reliability measures described above, we searched for population outliers. We defined an outlier for a specific measure as a participant whose score was more than three median average deviations (MAD) away from the population median. This technique yielded three outlier participants based on the Rating1-Rating2 test-retest reliability scores, and eight outlier participants based on the Rating1-Rating3 test-retest reliability scores. There was one outlier with respect to Certainty1-Certainty2, and six with respect to Certainty1-Certainty3. Seven of the outlier participants were caught by more than one filter, which left us with a set of 11 total outlier participants for Study 1. We excluded these participants from our reported analyses.

**Study 2**

Before testing our hypotheses, we performed a number of simple data quality checks. First, we assessed the test-retest reliability of value ratings. For each participant, we thus measured the pairwise linear correlation between first rating (Rating1) and second rating (Rating2), across items. We found that ratings were generally consistent (median Spearman’s rho = 0.803, p<0.001).

Next, we performed a similar assessment of the test-retest reliability of certainty reports. Before examining the certainty data, we first converted the qualitative reports to numbers (“not at all” = 1, “slightly” = 2, “somewhat” = 3, “fairly” = 4, “very” = 5, “extremely” = 6). For each participant we then measured the pairwise linear correlation between certainty for Rating1 (Certainty1) and certainty for Rating2 (Certainty2), across items. We found that certainty reports were generally consistent (median Spearman’s rho = 0.344, p<0.001), although much less so than value ratings.

Because rating certainty is a key variable for testing our hypotheses, we needed to be sure that participants responded meaningfully to the rating certainty question. An analysis of how certainty correlates with our other variables of interest would not be possible where there is insufficient variability in the certainty data. For this reason, we calculated a score for how much variance each participant had across certainty reports. The median certainty report variance was 0.971 and 0.818 for Rating1 and Rating2, respectively. We deemed that there were no participants who were obvious outliers based on this score.

Finally, we checked whether choices were consistent with pre-choice ratings. For each participant, we performed a logistic regression of choices against the difference in value ratings of the paired options. We found that the balanced prediction accuracy was beyond chance level (mean 77%), indicating participants were performing the choice task properly.

For each of the test-retest reliability measures described above, we searched for population outliers. We defined an outlier for a specific measure as a participant whose score was more than three median average deviations (MAD) away from the population median. This technique yielded three outlier participants based on the Rating1-Rating2 test-retest reliability scores. We excluded these participants from our reported analyses.

*Effect of confidence on choice consistency*

We then explored a step further, postulating that choice confidence should modulate choice consistency (often referred to as accuracy). The idea is that for high confidence choices, the DM would more consistently distinguish the items, relative to low confidence choices. We thus performed a similar logistic regression as we did in our main analysis (see Figure 6 in Results), for each participant, except this time the indicator represented high choice confidence (within-participant median split) instead of value certainty (choice = logistic[beta0 + beta1*dV + beta2*Ind*dV]). Under this model, balanced accuracy was also 77% (p<0.001). Again, there was no bias (mean beta0 = -0.028, p=0.466), and the inverse temperature parameter remained positive and significant (mean beta1 = 0.065, p<0.001). Notably, the regression coefficient for the interaction of value difference and the high confidence indicator (i.e., the increase in choice precision between low and high confidence trials) was positive and significant (mean beta2 = 0.088, p<0.001) (see Figure S1). We thus confirmed a common observation that choice confidence and choice accuracy are closely linked.

**Figure S1:** Across participants, the probability of choosing the option on the right increased as a function of the value estimate difference (right option – left option). In particular: choices that were made with low confidence (red curve, within subject median split) were more stochastic than choices that were made with high confidence (blue curve).
